# Current Surgical Management of Peri-Hilar and Intra-Hepatic Cholangiocarcinoma

**DOI:** 10.3390/cancers13153657

**Published:** 2021-07-21

**Authors:** Andrea Lauterio, Riccardo De Carlis, Leonardo Centonze, Vincenzo Buscemi, Niccolò Incarbone, Ivan Vella, Luciano De Carlis

**Affiliations:** 1 Department of General Surgery and Transplantation, Niguarda Ca’ Granda Hospital, 20162 Milan, Italy; andrea.lauterio@ospedaleniguarda.it (A.L.); riccardo.decarlis@ospedaleniguarda.it (R.D.C.); vincenzo.buscemi@ospedaleniguarda.it (V.B.); niccolo.incarbone@ospedaleniguarda.it (N.I.); ivan.vella@ospedaleniguarda.it (I.V.); luciano.decarlis@ospedaleniguarda.it (L.D.C.); 2 School of Medicine and Surgery, University of Milano-Bicocca, 20162 Milan, Italy; 3 Department of Surgical Sciences, University of Pavia, 27100 Pavia, Italy; 4 International Center for Digestive Health, University of Milano-Bicocca, 20162 Milan, Italy

**Keywords:** cholangiocarcinoma, intra-hepatic cholangiocarcinoma (i-CCA), peri-hilar cholangiocarcinoma (h-CCA), extended liver resection, associating liver partition and portal vein ligation for staged hepatectomy (ALPPS), portal vein embolization (PVE), trans-arterial chemoembolization (TACE), radioembolization, neoadjuvant chemoradiation, liver transplantation

## Abstract

**Simple Summary:**

The treatment of peri-hilar (h-CCA) and intrahepatic (i-CCA) cholangiocarcinoma is an evolving field in hepato-pancreato-biliary surgery. Continuous development of radiological and surgical techniques currently offers different treatment strategies, ranging from traditional hepatectomies to complex approaches involving preoperative portal vein embolization or associating liver partition and portal vein ligation for staged hepatectomy. Recent advances in perioperative chemo-radiotherapy have improved patient survival and have been incorporated into transplant protocols, yielding excellent results. We report a comprehensive review of current surgical and multimodal approaches to h-CCA and i-CCA treatment.

**Abstract:**

Cholangiocarcinoma accounts for approximately 10% of all hepatobiliary tumors and represents 3% of all new-diagnosed malignancies worldwide. Intrahepatic cholangiocarcinoma (i-CCA) accounts for 10% of all cases, perihilar (h-CCA) cholangiocarcinoma represents two-thirds of the cases, while distal cholangiocarcinoma accounts for the remaining quarter. Originally described by Klatskin in 1965, h-CCA represents one of the most challenging tumors for hepatobiliary surgeons, mainly because of the anatomical vascular relationships of the biliary confluence at the hepatic hilum. Surgery is the only curative option, with the goal of a radical, margin-negative (R0) tumor resection. Continuous efforts have been made by hepatobiliary surgeons in order to achieve R0 resections, leading to the progressive development of aggressive approaches that include extended hepatectomies, associating liver partition, and portal vein ligation for staged hepatectomy, pre-operative portal vein embolization, and vascular resections. i-CCA is an aggressive biliary cancer that arises from the biliary epithelium proximal to the second-degree bile ducts. The incidence of i-CCA is dramatically increasing worldwide, and surgical resection is the only potentially curative therapy. An aggressive surgical approach, including extended liver resection and vascular reconstruction, and a greater application of systemic therapy and locoregional treatments could lead to an increase in the resection rate and the overall survival in selected i-CCA patients. Improvements achieved over the last two decades and the encouraging results recently reported have led to liver transplantation now being considered an appropriate indication for CCA patients.

## 1. Introduction

Cholangiocarcinoma (CCA) accounts for approximately 10% of all hepatobiliary tumors and represents 3% of all new-diagnosed malignancies worldwide [1].

This rare tumor arises from the biliary epithelium and can develop at any level of the intra- and extrahepatic biliary tree; according to its location, CCA is generally classified as intra-hepatic, peri-hilar, or distal, representing three distinct entities in terms of biology, treatment options, and prognosis [2], with separate American Joint Committee on Cancer (AJCC) staging systems [3].

Intrahepatic CCA (i-CCA) accounts for 10% of all cases, perihilar CCA (h-CCA) represents two-thirds of the cases, while distal CCA accounts for the remaining quarter [4].

The surgical approach to distal CCA is usually represented by pancreatoduodenectomy [5]; as such, this subgroup of tumors is generally managed by pancreatic rather than hepatobiliary surgeons.

The following paper will focus on the surgical management and treatment of h-CCA and i-CCA.

## 2. Peri-Hilar CCA

Originally described by Klatskin in 1965 [6], h-CCA represents one of the most challenging tumors for hepatobiliary surgeons, mainly because of the anatomical vascular relationships of the biliary confluence at the hepatic hilum.

Surgery is the only curative option, with the goal of a radical, margin-negative (R0) tumor resection.

Considering its expansive and longitudinal growth pattern [7], extensive biliary infiltration and vascular invasion represent major challenges while attempting a radical surgery for h-CCA [8].

Continuous efforts have been made by hepatobiliary surgeons in order to achieve R0 resections, leading to the progressive development of aggressive approaches that include extended hepatectomies, associating liver partition and portal vein ligation for staged hepatectomy, pre-operative portal vein embolization, vascular resections, and liver transplantation.

Such complex strategies mandate a multidisciplinary work-up in highly specialized hepatobiliary and transplant units in order to optimize surgical and oncological outcomes. 

### 2.1. Diagnosis and Peri-Operative Management of h-CCA

The most frequent presentation of h-CCA is obstructive jaundice, associated with vague abdominal pain or discomfort, pruritus, or weight loss [9], with up to 10% of patients presenting with cholangitis [10].

Owing to the lack of early symptoms, a high percentage of patients are diagnosed with an advanced disease at presentation (vascular involvement; intra- or extra-hepatic metastases) [11].

A thorough pre-operative workup and appropriate peri-operative management are mandatory in order to achieve the best surgical and oncological outcomes and prevent postoperative complications [12]; the main goals of pre-operative work-up are to assess tumor burden and resectability, as well as grant proper biliary decompression of the future liver remnant.

#### 2.1.1. Pre-Operative Work-Up and Staging

The role of serum markers such as carbohydrate antigen (CA) 19-9 and carcinoembryonic antigen (CEA) as diagnostic tools is controversial; CA 19-9 has a variable sensitivity ranging from 33 to 93%, with a specificity of 67 to 98%, while CEA reaches a sensitivity of 33 to 84% and a specificity 50 to 87% [13]; despite that, serum CA 19-9 measurement represents a mandatory step in pre-operative work-up [14].

Novel biomarkers such a S100A4 calcium-binding protein have been associated with increased CCA invasiveness and potential for metastatization [15,16]; moreover, different methods for genetic characterization through liquid biopsy have been widely described in recent years as well [17], but their application in common clinical practice is still limited.

The main goal of pre-operative imaging in h-CCA is to determine tumor location and extension through the biliary tree, evaluate any vascular involvement at hepatic hilum, and rule out the presence of nodal or distant metastases, in order to allow an appropriate operative planning and staging [18].

The cornerstone of pre-operative radiological evaluation is represented by cross-sectional imaging-contrast-enhanced computed tomography (CT) and/or magnetic resonance imaging (MRI), that should be preferably performed before biliary drainage [14].

In their meta-analysis focused on radiological workup of peri-hilar cholangiocarcinoma, the Amsterdam group documented an 86% accuracy of CT for determination of tumor’s ductal extension, while the sensitivity and specificity were 89% and 92% for evaluation of portal vein involvement, 83% and 93% for hepatic artery involvement, and 61% and 88% for lymph node involvement, respectively [19].

MRI coupled with magnetic resonance cholangiopancreatography (MRCP) grants the same diagnostic accuracy of CT, with the advantage of a better visualization of the biliary tree [20,21]. Positron emitting tomography (PET) CT has a limited application owing to its low sensitivity in detecting tumor extension, but retains a significant role for detection of lymph node or distant metastases [22].

With the evolution of cross-sectional imaging, the role of endoscopic retrograde cholangiopancreatography (ERCP) during pre-operative workup of h-CCA has gradually evolved from a radiological diagnostic tool into an interventional procedure aimed to provide cytological specimens when pathological confirmation is required, with a 69–93% sensitivity range [23]. Single operator cholangioscopy offers a direct visualization for targeted biopsy of the tumor, representing an emerging tool with increased diagnostic accuracy compared with brushing techniques [24]. The application of endoscopic ultrasound with fine needle aspiration for cytological diagnosis of h-CCA also grants excellent diagnostic sensitivity [25], but could bear the risk of tumor seeding [26] and require specific expertise.

As almost 50% of potentially resectable surgical candidates will be deemed as unresectable at surgical exploration, staging laparoscopy has been proposed in order to avoid futile laparotomy; the all-cause yield of staging laparoscopy for uncurable disease has been reported between 27 and 45% [27,28], but its systematic use is controversial [29].

The principal staging systems for h-CCA are the Bismuth–Corlette classification; the Memorial Sloan Kettering Cancer Center (MSKCC); or Blumgart classification, the 8th AJCC staging system.

The Bismuth–Corlette classification focuses on proximal extent of biliary involvement, resulting in a highly intuitive tool; despite that, it does not provide any information concerning vascular involvement or liver lobar atrophy [30].

The MSKCC staging system provides a wider description of tumor extension, assessing crucial elements for pre-operative planning such as portal vein invasion and associated ipsi- or contralateral liver lobar atrophy [31].

The AJCC classification is highly detailed, but relies on pathological information, and its utility for pre-operative staging is quite limited [3].

A recent international consensus has proposed a new comprehensive staging system aimed to depict with high detail tumor extension and resectability, suggesting its general application in literature reports in order to compare oncological and surgical results from different series [32].

#### 2.1.2. Pre-Operative Biliary Drainage

Pre-operative biliary drainage in h-CCA surgical candidates represents a cornerstone of peri-operative care.

Obstructive jaundice hampers liver regeneration capability as it negatively affects mitochondrial function [33]; furthermore, jaundiced patients exhibit an impairment of the intestinal barrier function, enhancing bacterial translocation [34].

Several studies highlighted a higher number of postoperative complications in jaundiced patients undergoing liver resection [35,36,37,38], suggesting the benefit of pre-operative biliary drainage before surgery.

On the other hand, manipulation of the biliary tree has been associated with cholangitis and septic complications [37,39], with a potential increase of post-operative morbidity and mortality [39,40,41].

Given these assumptions, pre-operative biliary drainage is recommended in patients with a planned extensive resection with a small future liver remnant or who will undergo pre-operative portal vein embolisation [42,43], while it is mandatory in patients presenting with pre-operative cholangitis [44].

Biliary decompression can be achieved through percutaneous or endoscopic approaches, namely, percutaneous transhepatic biliary drainage (PTBD), endoscopic biliary stenting (EBS), or endoscopic nasobiliary drainage (ENBD).

A recent metanalysis compared the efficacy and safety of PTBD and EBS in resectable peri-hilar cholangiocarcinoma patients, and PTBD resulted in less conversion and lower rates of pancreatitis and cholangitis [45]; on the other hand, PTBD has been shown to bear the risk of seeding metastases [46].

Finally, ENBD has been associated with fewer complications compared with EBS [47], and is currently the preferred approach in Japanese centers [48], despite significant patient discomfort (Table 1).

### 2.2. Surgical Strategies for h-CCA

Liver resection represents the only potentially curative treatment for patients with resectable disease.

Different surgical strategies have been developed to achieve radical resection, and current approaches vary from standard to extended hepatectomies coupled to vascular resections and reconstructions.

#### 2.2.1. Importance of Resection Margin

R0 resection has been demonstrated to be a critical factor associated with improved survival, and thus is the primary goal of surgical therapy [49,50].

It has been observed histologically that cholangiocarcinoma spread along the bile duct extending beyond the palpable macroscopic tumor mass [51].

Reports suggesting that survival after R1 resection does not significantly differ from that after R0 resection are likely to result from the improper classification of cases with a too narrow resection margin as R0 instead of as R1 [52,53,54,55]. Therefore, Seyama et al. reported that survival after R0 resection with a tumor-free margin <5 mm did not differ from that after R1 resection [54]. Moreover, Ebata et al. recommended that a 1–2 cm margin is required to achieve the complete eradication of hCCA [56].

When a positive resection margin is diagnosed intraoperatively at the frozen section, further resection is recommended if technically feasible. While some authors have suggested that limited (<5 mm) further resection did not improve survival, Ribero et al. reported a significant survival benefit of secondary R0 resection [57,58].

In a more recent study, Ma et al. confirmed the importance of additional resection and highlighted that a margin >1 cm was associated with better survival [59].

#### 2.2.2. Hepatectomy for h-CCA

Although several authors have proposed bile duct resection alone for selected patients with Bismuth type I and II tumors, Lim et al. reported a higher R0 rate (100 vs. 73%) and 5-year survival (50 vs. 30%) with concomitant liver resection [60]. Jarnagin et al. suggested that hepatectomy should be performed in virtually all cases, being the only independent predictor of long-term survival in patients undergoing R0 resection [31].

Hepatectomy for h-CCA is an extensive procedure carrying a high risk of surgery-related complications. The constant need for bile duct resection and lymphadenectomy, as well as the concurrence of cholestasis and cholangitis, all account for a high mortality rate, ranging between 4 and 10% in recent reports [43,61,62]. Long- and short-term results are summarized in Table 2.

The type of hepatectomy should be determined based on the extent of biliary invasion and can be summarized roughly into four procedures: right or a left hemihepatectomy for patients with Bismuth type I to III tumors, and right or a left trisectionectomy for type IV tumors [63,64,65]. Right trisectionectomy generally allows more radical resections owing to the greater length of the left hepatic duct [66,67].

The main issue of major resection for hCCA is the large amount of healthy parenchyma that is resected along with the tumor at the expense of the functional liver remnant (FLR). In this context, Miyazaki et al. first suggested performing central hepatic resection as an alternative to major resection [68]. Although a selection bias may occur, as central resections tend to be reserved for tumors confined to the first-order hepatic ducts, different series have shown that postoperative survival was not compromised compared with major liver resection in selected patients [63,69,70,71].

Central hepatectomy (generally involving resection of segments 1, 4b, and 5) is technically more demanding compared with anatomic resection and frequently requires the anastomosis of multiple intrahepatic ductal openings [70,72].

#### 2.2.3. The Role of Caudate Lobectomy

Thanks to its central location, h-CCA has a high chance to invade the biliary branches or directly infiltrate the parenchyma of the caudate lobe [73].

Several analyses have demonstrated a survival benefit of aggressive surgical approach including extended liver resection and complete excision of the caudate lobe, which is currently widely accepted as standard surgical practice [49,74,75,76,77].

In a large retrospective analysis of 241 patients, Bhutiani et al. have recently questioned this practice, reporting that, although associated with a greater likelihood of R0 resection, caudate lobectomy did not increase overall or disease-free survival [78].

However, such results might have been biased by a too narrow resection margin in the cases classified as R0 [79].

#### 2.2.4. The Role of Portal Vein Embolisation and Associating Liver Partition and Portal Vein Ligation for Staged Hepatectomy

Portal vein embolization (PVE) induces hypertrophy of the FLR, potentially enhancing the safety of an extended hepatectomy.

However, the indications for PVE in patients with h-CCA are still not clear. In the largest studies available, the cutoff for FLR is 40%, sometimes in combination with indocyanine green clearance [80,81]. A recent review showed that, with this relatively conservative indication, postoperative mortality was <4% [82].

There are concerns about the use of associating liver partition and portal vein ligation for staged hepatectomy (ALPPS) in h-CCA surgery owing to the high morbidity and mortality rates [83,84].

Unlike patients with colorectal liver metastases, in most patients with h-CCA, bile is colonized as a result of perioperative biliary drainage, which may induce infective complications, leading to high morbidity and mortality rates. Moreover, bile leakage during or after the first stage of the procedure carries the risk of peritoneal dissemination [85].

In a recent case-control analysis of the international ALPPS registry, the mortality rate in the ALPPS group was twice as high as that among matched patients who underwent standard hepatectomy (48 vs. 24%) [86].

Several technical refinements have been proposed to lower the extent and invasiveness of stage 1 ALPPS. Boggi et al. have reported the interesting combination of laparoscopic stage 1 ALPPS and microwave ablation in a patient with h-CCA [87].

More recently, Sakamoto et al. developed a modified ALPPS procedure, in which transileocaecal portal vein embolization (TIPE) was combined with partial ALPPS, and successfully used this technique in three patients with h-CCA [88].

#### 2.2.5. Lymph Node Dissection in h-CCA

Lymph node (LN) metastases are a major negative prognostic factor for h-CCA, leading to an expected 5-year survival between 0 and 25% [89]. The hepatoduodenal and peripancreatic LNs act as first stations of the deep lymphatic drainage of the liver. The hepatoduodenal LNs drain into the celiac station and gastrocardiac station through the lesser omentum, while the peripancreatic LNs reach the superior mesenteric station [90]. Station number 12 (hepatoduodenal ligament) is the most frequently involved, and patients with positive LNs in other regional stations typically also have positive LNs in station number 12. Biopsy-proven metastases to LN stations outside the hepatoduodenal ligament as well as distant metastases are classical contraindications for nonresectability [91]. Nevertheless, several studies have reported a similar survival for patients with h-CCA with positive LNs limited to statin 12 and those with other positive stations [92,93]. Although the number of LNs retrieved for an adequate dissection is still debated, the positive LN ratio has recently been demonstrated to better stratify patients with LN metastases [89,94].

#### 2.2.6. Vascular Resections for Peri-Hilar Cholangiocarcinoma

Portal and arterial invasion were traditionally considered as absolute contraindications for surgical resection of hilar cholangiocarcinoma.

While the attitude of hepatobiliary surgeons towards peri-hilar cholangiocarcinoma surgery became more aggressive, vascular resection of the portal and arterial structures started to be considered as a concrete therapeutic option.

Portal vein resection was proposed as a standard procedure in the ‘no-touch’ approach described by Neuhaus et al. in 1999 [95]; the oncological benefit of this surgical approach was documented in a following paper by the same group, where patients approached with a ‘no-touch’ technique experienced a better overall survival compared with patients receiving a traditional hepatectomy (5-year overall survival: 58% vs. 29%) [66].

On the other hand, hepatic artery resection and reconstruction represents a technically challenging procedure [96], which can potentially increase postoperative morbidity without a clear oncological benefit [97].

Current evidence suggests that portal vein resection could increase R0 resections without affecting post-operative morbidity and mortality, while the role of arterial resection is still limited [96,97,98,99,100,101,102].

**Table 2:** Hilar cholangiocarcinoma, post-operative outcomes.

Author, Year	N of Patients	Morbidity (%)	Mortality (%)	Median Survival (mo)	5-Year Survival (%)
Farges et al. [43], 2013	366	69%	11%	-	-
Nagino et al. [63], 2013	574	57.3%	4.7%	-	32.5%
Yu et al. [102], 2014	238	18%	1%	-	17%
Furusawa et al. [64], 2014	144	86%	1,4%	-	35%
Tran et al. [65], 2019	257	66%	6%		19%
Franken et al. [62], 2020	178	77%	14%	66	38.2–43.7%^†^
Nagino et al. [67], 2021	787	-	1.6–2.6% ^†^	-	39–42% ^†^

Abbreviations: mo, months; N, number. ^†^ Results for right and left hemiepatectomy.

#### 2.2.7. Minimally Invasive Surgery for h-CCA

Most laparoscopic series are limited to bile duct resection and only a few of them reported extended hepatectomy and lymph node dissection [103,104].

The main recognized issues of laparoscopic resection for h-CCA are the lack of direct palpation of the hilar structures and the technical challenge of hepaticojejunostomy [105]. Giulianotti et al. reported the first robotic-assisted radical resection for h-CCA [106]. Xu et al. reported 10 cases of extended robotic resections; the robotic group compared unfavorably to open resection in morbidity (90 vs. 50%), and hospital expenditure, resulting in the conclusion that robotic surgery was not supported for the moment [107]. More recently, Li et al. have reported their single-center experience over 48 robotic procedures with 10.4% major morbidity and 72.9% R0 [108].

The same authors have noticed that the process of vessel skeletonization could lead to pseudoaneurysm formation, thus recommending a more cautious use of the electrothermal devices during robotic hilar dissection [107,108].

### 2.3. Multimodal Treatment Strategies for h-CCA

#### 2.3.1. Neoadjuvant Chemotherapy

Currently, there is no consensus on the role of neoadjuvant treatment for h-CCA, as the lack of a clear definition for borderline resectable tumors, as in the field of pancreatic ductal adenocarcinoma, restricts the use of neoadjuvant therapy before liver resection for h-CCA. Matsuyama et al. recently proposed to define borderline resectability as the coexistence of both regional lymph node metastasis and vascular invasion and demonstrated that these cases had no better outcomes than the unresectable ones [109].

Several case series biased by small sample size and a poor distinction between hilar and distal cholangiocarcinoma have reported the potential effectiveness of neoadjuvant chemoradiotherapy in enhancing R0 resection rate [110,111]. In a retrospective study by McMasters et al., nine patients unresectable at presentation (five with h-CCA and four with distal cholangiocarcinoma) underwent neoadjuvant chemoradiation; all the patients responded to treatment, and the overall rate of R0 surgery was 100%, with no recurrences in the h-CCA group [112].

Several other retrospective series have been subsequently published [113,114,115], which are shown in Table 3. A meta-analysis has recently confirmed that chemotherapy plus radiotherapy has the potential to provide high conversion rates of advanced and unresectable h-CCA to surgical resection [116]. However, the interpretation of these data should be cautious given the likelihood of publication bias. The phase II trial of the NACRAC study, aimed at evaluating the pathological curability and survival after neoadjuvant gemcitabine and external beam radiation, is currently in progress [117].

Conversely, the use of external beam radiation therapy alone seems to result in a limited R0 resection rate [116,118].

An alternative, relatively new treatment strategy is neoadjuvant photodynamic therapy (PDT). In a pilot study by Wiedman et al. including seven unrespectable h-CCA patients, neoadjuvant PDT led to R0 resection in all patients [119].

#### 2.3.2. Adjuvant Chemotherapy

Although still controversial, there is increasing evidence that adjuvant treatment in the form of either chemoradiation or systemic chemotherapy may improve local control and survival in patients with resected h-CCA.

Kang et al. reported a survival benefit with adjuvant 5-fluorouracil-based chemotherapy combined with radiotherapy in patients with positive lymph nodes [120].

A recent multivariate analysis has confirmed these results on a U.S. multicenter cohort of 249 patients [121].

## 3. Intra-Hepatic CCA

Intrahepatic cholangiocarcinoma (i-CCA) is an aggressive biliary cancer that arises from the biliary epithelium proximal to the second-degree bile ducts.

The incidence of i-CCA is increasing worldwide, and surgical resection is the only potentially curative therapy, even if the 5-year overall survival is poor, ranging between 15% and 45% owing to several risk factors like multifocal disease, margin status, vascular infiltration, and lymph nodes’ involvement [4,122,123,124,125,126,127].

Long- and short-term outcomes of surgical resection for i-CCA are summarized in Table 4.

A complete surgical resection is feasible only in 30–40% of i-CCA patients, often because of late diagnosis [128].

An aggressive surgical approach, including extended liver resection and vascular reconstruction, and a greater application of systemic therapy and locoregional treatments could lead to an increase in the resection rate and the overall survival in selected i-CCA patients.

### 3.1. Incidence and Risk Factors

The incidence of i-CCA has increased dramatically in recent years, probably because of improvements in differential diagnoses related to better imaging, molecular diagnostics, and pathology accuracy [129].

At the same time, mortality rates from i-CCA have shown a growing trend, predominantly justified by a better disease classification [130].

The clinical outcome of patients with i-CCA is unfavorable, with 5-year survival ranging from 15% to 45%, depending on tumor stage [122,131].

Several risk factors are associated with i-CCA: primary sclerosing cholangitis (PSC), inflammatory bowel diseases, fluke infections of the biliary tract, hepatitis B virus (HBV) and hepatitis C virus (HCV) infections, cirrhosis, diabetes, obesity, alcohol, and tobacco. However, the majority of i-CCA patients have no underlying liver disease [132,133].

### 3.2. Classification

i-CCA exhibits different growth patterns and is classified as mass-forming, periductal infiltrating, or mixed types, based on the Liver Cancer Study Group of Japan (LCSGJ) classification [134]. The LCSGJ also defines an intraductal growth type, which is rare and associated with favorable prognosis.

The mass-forming is the most common type, characterized by an intraparenchymal mass with distinct borders, while the periductal-infiltrating type causes tumor infiltration along the bile ducts. Some studies show worse survival after resection of the periductal-infiltrating type compared with the mass-forming type, whereas other reports show no difference in survival [135,136,137].

Recently, histologic studies have divided i-CCA into large duct and small duct subtypes, based on macroscopic growth patterns.

### 3.3. Diagnosis

Owing to peripheral liver growth of i-CCA and to the normal hepatic function, patients with i-CCA often present non-specific symptoms such as vague right upper quadrant pain, weight loss, and fatigue, while jaundice is less common compared with h-CCA patients (15–16% of cases).

i-CCA is most commonly found in asymptomatic patients who undergo imaging for abnormal liver enzymes or a reason unrelated to i-CCA.

Tumor markers like CA 19.9 and CEA are limited for diagnosis for their low sensitivity for early stage i-CCA.

As a result, i-CCA is frequently diagnosed as an advanced disease with bulky locoregional involvement and/or distant metastases and a complete surgical resection is only feasible in 30–40% of patients [128].

On CT scans, i-CCA shows the typical appearance of a hypodense hepatic mass in the unenhanced phase with irregular margins, peripheral rim-enhancement in the arterial phase, and progressive hyperattenuation on venous and delayed phases.

In some cases, such as small mass-forming type or in the case of underlying liver cirrhosis, i-CCA can radiologically mimic hepatocellular carcinoma (HCC).

Liver biopsy can provide differential diagnosis, especially in the case of atypical liver lesions, but tumor seeding could occur in the case of percutaneous biopsy.

Moreover, the histological aspect of i-CCA is similar to metastatic adenocarcinoma arising from the gastrointestinal tract; as such, endoscopic examination should be recommended in the preoperative assessment; in this setting, whole body CT scan or PET are mandatory for staging [128].

### 3.4. Surgical Treatment

Liver resection represents the only potentially curative treatment for patients with resectable disease, although most patients are not candidates for surgery because of the presence of advanced disease at diagnosis.

The overall survival and disease-free survival depend on tumor status at the time of diagnosis and several risk factors.

#### 3.4.1. Importance of Resection Margin

The influence of margin status on long-term outcome of patients undergoing liver resection for i-CCA remains controversial.

Several studies reported R0 resection as a significant predictor of survival and recurrence [138,139], while others suggested that margin status is not a significant predictor of outcomes [140,141].

The impact of margin width on long-term outcomes is still debated; Ribero et al. demonstrated that survival is not influenced by the width of a negative resection margin [138], while Farges et al. and Spolverato et al. noted that a tumor-free margin of 0.5 cm (in negative lymph nodes cases) and 1 cm, respectively, are independent prognostic factors for long-term survival [140,142]. Conversely, Watanabe et al. demonstrated that a surgical margin width of >1 cm in negative lymph nodes i-CCA patients is associated with better overall survival [143].

These conflicting results are probably due to stronger negative prognostic factors, such as lymph nodes’ involvement [138].

To date, available evidence supports the recommendation that hepatic resection with negative margins should be the goal of surgical therapy in potentially resectable i-CCA. Anatomical liver resection should be preferred to atypical resection, resulting in better overall survival and a lower recurrence rate [144].

#### 3.4.2. Extended Liver Resections

Extended liver resection with or without vascular or biliary reconstructions is often required to obtain R0 margins, especially in cases of voluminous lesions or multifocal tumors, which account for 50–70% of all i-CCAs cases, despite a higher rate of R1 resection related to this surgical approach compared with minor resections [142,145].

Similarly to other liver tumors, an extended liver resection must grant an adequate FLR; in i-CCA patients with normal liver function, an FLR of 25–30% is sufficient to avoid post-operative liver failure, while patients with chronic hepatic disease or portal hypertension require at least 40% of FLR.

Different strategies could be employed in order to improve FLR while planning an extended resection, such as PVE or ALPPS.

PVE is a relatively safe procedure, it allows FLR hypertrophy after a median of 4 weeks, and is currently considered the gold-standard strategy to induce FLR hypertrophy and lower the risk of post-hepatectomy liver failure [146].

Despite that, PVE shows several drawbacks: FLR growth could not be sufficient to avoid post-hepatectomy liver failure, especially in chronic liver diseases where FLR increased by 10% versus 30–40% in healthy livers [82]; the tumor could spread during the waiting regeneration time in about 40% of cases [147]; and the procedure could not be feasible for tumor localization (es. centrohepatic tumors).

ALPPS provides a fast hypertrophy of FLR (74% after a median of 9 days), avoiding the risk of tumor progression compared with PVE [147].

However, ALPPS presents high morbidity and mortality in comparison with conventional major hepatectomy. A recent international multicenter study considering 102 advanced i-CCA patients treated with ALPPS showed an overall morbidity after second stage of 77% (Clavien–Dindo grades 3b to 5 of 41%) and a 90-day mortality of 21% (despite that the 90-day mortality of latest cases dropped by 7%, probably owing to a technical improvement).

These data are consistent with other studies about ALPPS performed in HCC and h-CCA patients [86,148].

It must be noted that lower FLR/BW (body weight) and FLR/TLV (total liver volume) were the only significant risk factors for Clavien–Dindo grade >3b and 90-day mortality in multivariate analysis, respectively.

When a conventional major hepatectomy cannot provide an adequate FLR, ALPPS procedure significantly improves overall survival compared with palliative chemotherapy, despite a considerable morbidity and mortality [149].

Better patient selection and more favorable FLR/BW and FLR/TLV could improve the results and include this approach in current practice. 

Further studies are necessary to directly compare ALPPS and PVE with or without bridge systemic chemotherapy.

#### 3.4.3. Multifocal Disease

Multifocal disease deserves a dedicated mention: patients with multiple lesions usually develop early tumor recurrence (4.5 months vs. 9 months, as for i-CCA advanced stage). Non-surgical treatments such as chemoembolization or radioembolization seem to offer the same long-term results as surgery [150].

Although the current staging system proposed by the American Joint Committee on Cancer, AJCC 8th edition, classifies liver metastasis in i-CCA patients as early stage (in the absence of lymph node metastasis), multifocal liver disease is actually considered as one of the most important negative prognostic factors, independent of nodal tumor spread, thus surgical indication is questionable [151,152]. Recently, Lamarca et al. proposed a modified version of current AJCC guidelines, including i-CCA N0 patients with multifocal liver disease in a sub-metastatic group (i-CAA patients with extrahepatic metastasis have a worst prognosis) [153].

#### 3.4.4. Lymph Nodes

Despite the negative prognostic value of metastatic lymph nodes, extensive lymphadenectomy is not routinely performed in the case of macroscopically non-suspicious lymph nodes [154], so that data concerning lymph nodes status are available for only 49% of i-CCA patients according to the American National Cancer Institute database [155].

Current data suggest that the incidence of lymph nodes metastases ranges from 30% to 40% among patients undergoing lymphadenectomy [128,145].

Several authors demonstrated that routine lymphadenectomy for i-CCA patients does not provide survival benefits [156,157,158], whereas other studies highlighted better long-term outcomes in patients with node negative i-CCA [159].

Although there is no evidence supporting the therapeutic role of lymphadenectomy in i-CCA patients, current guidelines recommend lymphadenectomy of porta hepatis (including inferior phrenic and gastrohepatic for left-sided lesions and periduodenal and peripancreatic for right-sided i-CCAs) in order to achieve a complete staging and for the crucial prognostic role of nodal involvement [3,160].

Lymphadenectomy should also be performed in very small i-CCA (2–3 cm), as the positive nodes ratio reaches 20% to 30%.

Anyway, the relationship between tumor stage and the prevalence of lymph nodes’ metastases has not been clearly demonstrated [161,162].

Considering that locoregional nodal metastases is one of the most negative prognostic factors, a surgical resection could be contraindicated in these patients.

Kizy et al. highlighted that surgical resection for patients with positive nodes may not improve survival compared with chemotherapy alone, despite that only one half of resected patients underwent adjuvant chemotherapy [163].

Conversely, Tran et al. noted that surgical resection plus chemotherapy in i-CCA patients with nodal metastasis resulted in a median survival of 23 months versus 13 months for patients who underwent non-operative treatment [164].

In a recent large series (1013 i-CCA patients with positive nodes on pre-operative imaging), liver resection plus chemotherapy was associated with improved survival (22.5 months) compared with chemotherapy (11.9 months) or resection alone (12.4 months) [164,165].

As a result, i-CCA resectable patients with clinical suspected nodes should not be excluded from a surgical approach.

Considering the suboptimal accuracy of CT or PET scans in determining metastatic nodes, pre-operative lymph nodes’ enlargement (>1 cm) seems not be a significant prognostic factor; to date, the most appropriate pre-operative work-up for patients with suspected lymph nodes metastasis has not yet been determined [166].

In the case of multiple “worrisome features” such as clinical nodes, high CA 19.9 levels, vascular invasion, large i-CCA, or multifocal disease, a staging laparoscopy could be suggested to eventually detect an advanced disease contraindicating liver resection.

#### 3.4.5. Minimally-Invasive Surgery for i-CCA

Despite a growing interest in minimally invasive approaches for liver tumors, in the recent Southampton Guidelines consensus on laparoscopic liver surgery, the role of laparoscopy in i-CCA was not assessed, probably owing to a significant lack of data [167]. The adoption of laparoscopy for i-CCA has been relatively delayed because of several factors like challenging resection for central i-CAA, extrahepatic resections, tumor vascular infiltration, and the need to perform a lymphadenectomy. Nevertheless, the available series of laparoscopic resection for i-CAA demonstrated better short-term outcomes and comparable long-term outcomes when compared with the laparotomic approach [168]. However, further studies are necessary to establish the real benefits of laparoscopy because the minimally invasive approach is frequently applied for small tumors and the lymphadenectomy is not so frequently performed [169,170]. The robotic surgery could offer a significative advantage for i-CAA, helping the surgeon to perform the lymphadenectomy or complex hepatic resection in the case of vascular infiltration, thanks to articulating instruments with seven degree of freedom [171].

### 3.5. Systemic Treatments

The current evidence concerning systemic treatments for i-CCA is summarized in Table 5.

Adjuvant chemotherapy is not standardized for i-CCA patients. The phase III BILCAP study showed improved overall survival after adjuvant capecitabine-based treatment, even if overall survival did not reach statistical significance in the intention-to-treat analysis [172].

The Prodige-11 trial compared adjuvant gemcitabine plus oxaliplatin (GEMOX combination) versus observation in resected biliary tract cancer, but no benefits were found [173].

To date, the National Comprehensive Cancer Network (NCCN) suggests adjuvant chemotherapy after curative surgery of cholangiocarcinoma with different regimens tailored on lymph nodes and margin status, recommending chemotherapy with gemcitabine and cisplatin for unresectable or advanced i-CCA, as highlighted by ABC-02 trial (overall survival of 11.7 months with gemcitabine and cisplatin, compared with 8.2 months with gemcitabine alone) [174]. A recent meta-analysis showed that adjuvant chemotherapy is associated with improved overall survival and should be considered in patients with i-CCA following curative resection, particularly for patients with advanced disease [175].

There are currently no RCTs analysing the role of neoadjuvant chemotherapy in unresectable i-CCA patients.

The use of neoadjuvant chemotherapy is not frequent in clinical practice, mainly because of poor tumor response to medical treatment, and upfront surgery (including extensive liver resection) is still considered the best treatment as long as it could grant oncological radicality (R0 resection).

However, identification of negative prognostic risk factors such as positive nodes, vascular invasion, multifocal disease, or large tumor size associated with an increased risk of R1 resection led to a more frequent employment of medical treatment for tumor downstaging (only 20–40% of ICC cases are resectable at the diagnosis) [152]. The rationale for medical treatment is not only to achieve resectability, but also to gain a better R0 rate and evaluate tumor biology, avoiding unnecessary surgical treatment. 

In well selected cases, neoadjuvant chemotherapy followed by surgery could increase survival in advanced i-CCA patients instead of upfront surgery or chemotherapy alone [176,177,178].

The role of radiation therapy for resected i-CCA patients is not well defined.

A recent retrospective study demonstrated that radiotherapy did not provide a survival benefit in patients with positive resection margins and negative lymph nodes [179].

Current guidelines do not suggest systematic use of radiation therapy after surgery.

A recent RCT showed that high-dose radiotherapy improved local control and overall survival in unresectable i-CCAs [180].

### 3.6. Locoregional Treatments

Unresectable i-CCA generally leads to death for tumor-related liver failure, even in the case of extrahepatic distant metastasis (i.e., lymph nodes, peritoneal, lung), as tumor growth involves parenchymal loss and liver failure due to vascular and biliary obstruction [180]. Locoregional treatments could represent a palliative option to delay liver failure.

A retrospective cohort study of 155 unresectable i-CCA patients reported a significantly longer overall survival in patients treated with TACE (12.2 months) compared with a non-TACE treated cohort (3.3 months) [181], and other studies support these findings, showing a median overall survival of up to 12–13 months [182,183].

A systematic review by Al-Adra et al. summarized the clinical evidence available for the efficacy and toxicity of radioembolization, reporting a median overall survival of 15.5 months, with seven patients downstaged for surgery [184].

Rayar et al. showed a median overall survival of 19.1 months for patients undergoing surgery after downstaging by radioembolization combined with systemic chemotherapy (8 of 45 i-CCA unresectable patients) [185].

Another phase 2 clinical trial analyzing the effects of SIRT plus chemotherapy for unresectable i-CCA documented an overall survival of 75% at 12 months and 45% at 24 months, with 22% of patients downstaged to surgical intervention, with 20% achieving R0 surgical resection [186].

In summary, despite the lack of RCTs, the available literature supports the use of locoregional treatments for patients with unresectable i-CCA.

TACE is probably the more effective and used treatment for local control and downstaging, but radioembolization is increasingly utilized, especially in the case of multinodular disease [187].

**Table 5:** Perioperative systemic and locoregional treatment for i-CCA.

Author, Year	Study Design	Treatment Timing	Type of Treatment	Main Results	Secondary Endpoints
Kato et al. [176], 2013	Retrospective	Neoadjuvant	Gemcitabine	45% 5-year OS after downsizing CT	CT enabled surgery in 36.4% of patients
Le Roy et al. [177], 2018	Retrospective	Neoadjuvant	CT + surgery (unresectable i-CCA) vs. surgery alone (resectable i-CCA)	No difference 3- and 5-year OS Higher R0 resection rate in surgery group	No difference in terms of intra- and post-operative results
Yadav et al. [178], 2019	Retrospective	Neoadjuvant	CT + surgery vs. surgery + CT	Longer OS in neoadjuvant group (40.3 vs. 32.8 months) Longer 5-year OS in neoadjuvant group (42.5% vs. 31.7%)	Higher R0 resection rate in neoadjuvant group
Hammad et al. [179], 2016	Retrospective	Adjuvant	Surgery + RT vs. surgery alone	No difference in OS after R0 resections RT after R1/R2 resections was associated with improved OS (39.5 vs. 21.1 months)	
Tao et al. [180], 2016	Prospective	Palliative treatment	RT (unresectable i-CCA)	3-year OS rate was 44%	Higher RT doses (80.5 Gy) correlated with better 3-year OS compared with lower doses (73% vs. 38%)
Park et al. [181], 2011	Retrospective	Palliative treatment	TACE vs. supportive treatment in unresectable i-CCA	12-months survival vs. 3-months in TACE group, even for patients with extrahepatic metastasis	
Rayar et al. [185], 2015	Retrospective	Neoadjuvant	Radioembolization	Median disease-free survival of 19.1 months	TARE enabled surgery in 80% of patients (8/10)
Edeline et al. [186], 2019	RCT	Neoadjuvant	Radioembolization + CT	2-year OS rate of 45% 2-year progression-free survival rate of 30%	TARE enabled surgery in 22% of patients (9/32)
BILCAP Trial [172], 2019	RCT	Adjuvant	Capecitabine	No improvements in OS when used as intention-to-treat	May improve overall survival when used as adjuvant therapy (53 vs. 36 months)
Prodige-12 Trial [173], 2019	RCT	Adjuvant	Gemcitabine plus oxaliplatin vs. surveillance	No benefit for OS	

Abbreviations: CT, chemotherapy; OS, overall survival; RCT, randomized controlled trial; RT, radiotherapy; TACE, trans-arterial chemoembolization; TARE, trans-arterial radioembolization.

### 3.7. Management of Disease Recurrence

The 5-year recurrence free survival ranges from 2% to 39% after curative resection for i-CCA [151].

The most common recurrence site is the liver (82.7%), especially within 24 months from resection, while recurrence after 24 months is mostly isolated to an extrahepatic site such as lymph nodes, lungs, or to a less common site like bone, skin, or chest wall [188]. Systemic chemotherapy is the common treatment of disease recurrence of i-CCA after surgery.

Locoregional treatment as TACE, SIRT, or percutaneous ablation could play a role for recurrence disease control, but available studies are scarce in the literature.

Spolverato et al. reported a modest benefit in median survival for patients who underwent repeated surgery over those who received percutaneous ablation or intra-arterial therapy [189].

Zhang et al. reported a significant survival benefit of repeated resection for large tumor (>3 cm), and such results were confirmed by Bartsch et al. in a small series comparing repeated hepatectomy to locoregional treatments [190,191].

If a R0 resection is achievable, a repeated hepatectomy in selected patients with recurrent i-CCA is reasonable.

## 4. Liver Transplantation for h-CCA and i-CCA

### 4.1. Preamble

Liver transplantation (LT) for unresectable CCAs has been met with skepticism on account of very high recurrence rates, as well as the poor overall results obtained in the late 1990s [192,193,194,195,196].

Improvements achieved over the last two decades and the encouraging results recently reported have led to liver transplantation now being considered an appropriate indication for CCA patients.

Given the organ shortage, the main concern when offering a transplant to these patients is the impact on other patients on the waiting list.

However, advances in the management of HCV seem to have led to fewer HCV patients needing LT, with a potential increase in the organ pool, albeit with differences between countries [197].

In addition, living donor liver transplantation (LDLT) has been increasingly resorted to for patients with CCA to address the problem of organ shortage, reducing prolonged waiting times and avoiding recipient patient morbidity due to sepsis and the consequent need for biliary interventions [198].

### 4.2. Liver Transplantation for h-CCA

As previously exhaustively reported, current 5-year survival after curative surgery for h-CCA rarely exceeds 40% even in high volume centers and in very selected patients.

These poor outcomes following liver resection prompted consideration of LT as a curative approach for h-CCA patients on the basis that it would allow complete resection of a locally unresectable tumor, resolve insufficient hepatic function issues, and cure the underlying liver disease in a setting of liver cirrhosis or PSC.

However, the few studies initially published failed to show promising results after liver transplantation for h-CCA, with 3- and 5-year survival never exceeding 40% and 30%, respectively, and with very high tumor recurrence rates [194,195,196].

At the turn of the century, the experiences of the University of Nebraska and the Mayo Clinic triggered a revolution in the field.

The Mayo Clinic’s multimodal treatment approach of preoperative/neoadjuvant chemoradiation followed by transplantation reported prolonged disease-free survival in strictly selected patients with early unresectable h-CCA and solitary tumors of <3 cm, without evidence of lymph node involvement [199].

The first eleven patients received a combination of external beam radiation (40–45 Gy) followed by trans-catheter radiation (20–30 Gy) with iridium wires, and intravenous 5-fluorouracil administered for chemosensitization during radiation therapy prior to transplantation.

The encouraging results have been continuously updated over the years, and in 2004, an 82% 5-year survival rate was reported for 24 patients undergoing the so-called «Mayo Clinic protocol» [200].

In the following years, further studies from the same team compared 26 patients undergoing only liver resection with 38 patients undergoing liver transplantation after neoadjuvant therapy; the overall survival at 1, 3, and 5 years in the two groups was 82–48%, 21–92%, and 82–82%, respectively. Importantly, higher post-transplant recurrence was observed in the liver resection group compared with the transplanted patients (13% versus 27%) [201].

Improvements over the last two decades in the management of unresectable h-CCA, together with the results of liver transplantation following neoadjuvant chemoradiation, have led some centers to consider liver transplantation as a viable curative option for h-CCA. This has had important implications in terms of patient allocation and prioritization criteria, especially where allocation is based on a model for an end stage liver disease (MELD) driven score.

In 2006, the University of Nebraska proposed that, in a PSC setting, patients with unresectable h-CCA should receive MELD score exception points, which afforded better liver graft allocation [202].

The appropriateness of this MELD exception hypothesis was investigated and confirmed in 2012 by an American multicenter analysis including 287 patients with h-CCA treated with neoadjuvant therapy followed by transplantation from 1993 to 2010 in 12 large-volume transplant centers in the United States [203]. The study confirmed the combination therapy to be highly effective in a rigorously selected patient group. The 65% recurrence-free survival rate at 5 years reported, along with a dropout rate after 3.5 months of therapy of 11.5%, validated the appropriateness of granting MELD exception points and consequent waiting list prioritization.

To date, United Network of Organ Sharing (UNOS) and Organ Procurement and Transplant Network (OPTN) criteria require the h-CCA to be classified as unresectable after completion of a standardized neoadjuvant treatment protocol for being eligible for MELD exception points [202,204]. 

In 2012, Darwish Murad S et al. reviewed all patients with unresectable h-CCA treated with neoadjuvant chemoradiation before LT between 1993 and 2010; among 199 patients enrolled, 62 dropped out while 137 underwent LT [205].

Identified predictors of pre-transplant drop-out were represented by a CA19-9 ≥500 U/mL (hazard ratio (HR): 2.3; *p* = 0.04), a mass >3 cm (HR: 2.1; *p* = 0.05), malignant brushing or biopsy (HR: 3.6; *p* = 0.001), and a MELD score of ^3^20 (HR: 3.5; *p* = 0.02). Predictors of posttransplant recurrence were the following: elevated CA19-9 (HR: 1.8; *p* = 0.01), portal vein involvement (HR: 3.3; *p* = 0.007), and evidence of residual tumor on the explanted liver (HR: 9.8; *p* < 0.001.) In contrast, PSC, age, history of cholecystectomy, and wait-time were not found to be independent predictors of patient drop-out [203].

Pathological evaluation of the response to the neoadjuvant therapy on the explanted liver allows prediction of overall survival. Recipients showing minimal response to the therapy (percentage of residual viable tumor ≥30%) should undergo adjuvant chemotherapy (gemcitabine and cisplatin) up to ten months after transplantation, and immunosuppressive therapy should be shifted from calcineurin inhibitors (CNIs) to sirolimus/everolimus [204,206].

Other studies have reported further predictors affecting long-term survival. Robles et al. report stage III and stage IVA to be independent risk factors for poor prognosis in a multivariate analysis (*p* < 0.01) [195].

In their most recent analysis, Dondorf et al. report that pre-transplant CA 19-9 ≥500 U/mL, pre-transplant PTBD positioning, positive lymph nodal status, and advanced UICC stage were significant risk factors for overall survival [195,207].

Whether resection is more effective for h-CCA than transplantation after adjuvant chemotherapy is still under investigation. A multicenter study recently confirmed substantially higher survival rates for transplantation compared with resection in h-CCA patients falling within the transplantation criteria (<3 cm, lymph node negative disease) [208]. Forty-one transplanted patients showed improved overall survival compared with 191 patients treated by curative-intent resection (3 years: 72% vs. 33%; 5 years: 64% vs. 18%; *p* < 0.001).

In addition, liver transplantation was found to be associated with improved overall survival compared with liver resection in lymph node negative, non-PSC patients with tumors <3 cm: 3 years: 54% vs. 44%; 5 years: 54% vs. 29%; *p* = 0.03.

In 2016, a retrospective study from the European Liver Transplant Registry (ELTR) compared 28 patients included in the “Mayo protocol” with 77 outside the protocol receiving transplants for h-CCA between 1990 and 2010 [209]. The study showed an actuarial 5-year survival for the entire group of 105 transplanted patients of 32%. The patients falling within the Mayo Clinic criteria showed significantly better survival compared with patients outside the criteria (5-year survival of 59% compared with 21%; *p* = 0.001). The study confirmed once again that improved survival after transplantation for h-CCA can be achieved if the Mayo Clinic’s strict selection criteria are adopted.

A recent single-center European experience of 26 h-CCA transplanted patients following neoadjuvant chemoradiotherapy confirmed that achieving a complete pathological response conferred a significant benefit in terms of patient survival (median survival of 83.8 months vs. 20.9 months; *p* = 0.036) [210].

Long-term outcomes after LDLT for h-CCA are comparable with those of h-CCA patients transplanted with whole liver allograft [198]. Tan et al. compared 74 patients undergoing LDLT for h-CCA with 173 for other indications. The overall survival after LDLT in patients with h-CCA with underlying PSC at 1, 3, 5, and 10 years was superior (89.8%, 75.9%, 75.9%, and 73.2%, respectively) to that of patients without PSC (75%, 58%, 47.5%, and 35.2%, respectively).

In Italy, the indication for LT in patients with h-CCA is still under investigation in terms of organ allocation policy and recipient selection criteria. Nonetheless, the ongoing allocation policy proposed in 2016 allows the use of 5% of all available grafts for oncological indications other than HCC, which also include h-CCA [211].

The current evidence on LT for h-CCA is summarized in Table 6.

### 4.3. Liver Transplantation for i-CCA

Historically, from the mid 1990s, liver transplantation for i-CCA has been a contraindication in light of the poor (below 25%) 5-year overall survival reported [192,193,212].

The indication for liver transplantation for i-CCA is quite recent compared with unresectable h-CCA. This is especially true in the case of locally advanced unresectable i-CCA in the setting of neoadjuvant therapies. Response to these treatments can be considered a surrogate for assessing disease aggressiveness, and lasting stable response as a yardstick for selecting patients for transplantation.

Two multicenter studies recently reported favorable outcomes after liver transplantation for i-CCA in the setting of liver cirrhosis. Transplanted patients with uninodular i-CCA of less than 2 cm had similar overall survival rates compared with HCC patients with similar characteristics [213].

More recently, another study of a group of highly selected transplanted patients with well-differentiated i-CCA (<2 cm) showed a 5-year actuarial survival rate of 65% versus 45% for those with more advanced i-CCA (>2 cm), with a cumulative recurrence rate at 5 years of 18% versus 61%, respectively [214].

In 2018, a prospective case series confirmed encouraging results in selected patients with locally advanced i-CCA and disease stability after neoadjuvant therapy. Six patients with more than 6 months of radiological response or disease stability while on the waiting list after a gemcitabine-based neoadjuvant regimen (gemcitabine–cisplatin or gemcitabine–capecitabine) underwent liver transplantation [215].

One year, 3-year, and 5-year overall survival was 100% (95% CI 100–100), 83.3% (27.3–97.5), and 83.3% (27.3–97.5), respectively. Three patients developed recurrent disease at a median of 7.6 months (IQR 5.8–8.6) after transplant, with a recurrence-free survival of 50% (95% CI 11.1–80.4) at 5 years.

Radioembolization with Y90 to downstage unresectable i-CCA allowing surgical resection is another option under investigation, and could represent a bridging therapy to transplantation in selected patients [184,216].

### 4.4. Technical Considerations

In order to exclude the circa 20% of patients with lymph nodes and/or peritoneal disease after neoadjuvant therapy, pre-transplant staging is strongly recommended, preferably with minimally invasive exploration.

Given the exceptional circumstances of this transplant surgery, hepatic hilum dissection should be minimal and carried out as low as possible, while the bile duct should be sectioned as close to the duodenal margin as possible.

Recipient bile duct frozen section examination is mandatory to evaluate the margin, especially in a PSC setting.

Vascular injuries from radiation may require an aortic interposition jump graft for arterial reconstruction as well as portal reconstruction with the interposition of a vein graft. These technical issues are particularly challenging during LDLT for h-CCA compared with LDLT for other indications. The higher incidence of vascular complications in this setting was recently reported [198,204].

However, neither vascular nor biliary complications are associated with significantly worse outcomes after LDLT for h-CCA [198].

In this transplant setting, biliary reconstruction with a Roux-en-Y choledocho-jejunostomy should be considered mandatory [217].

## 5. Current Challenges and Future Perspectives

Surgical management of h-CCA represents one of the main challenges in hepatobiliary surgery, and the type and extent of liver resection that could be performed is still a matter of debate. Continuous improvements of surgical technique coupled to better peri-operative care could extend the traditional limits of resectability without compromising postoperative outcomes; for example, vascular invasion does not represent an absolute contraindication for surgical resection in specialized centers, and such an attitude actually challenges the current staging systems for h-CCA, which should undergo further refinements. Similarly, other complex approaches including PVE or ALPPS have been progressively explored in order to expand the tumor resectability, and their application is constantly growing worldwide. The introduction of minimally invasive laparoscopic and robotic approaches represents another stimulating challenge for both h-CCA and i-CCA, although their feasibility is still being explored.

Deepest knowledge of tumor biology is another key point that could lead to better patient and treatment selection in both h-CCA and i-CCA: novel biomarkers as well as liquid biopsy are currently under investigation, and their application in clinical practice will probably play a crucial role in patient stratification and better treatment selection.

Considering its encouraging results, the role of liver transplantation for both resectable and unresectable diseases will represent another challenge that will face hepatobiliary and transplant surgeons in the next future: a nationwide French multicenter randomized, intent-to-treat designed study (TRANSPHIL/NCT02232932; Liver Resection versus Radio-Chemotherapy-Transplantation for Hilar Cholangiocarcinoma) evaluating the survival of patients with resectable disease treated with neoadjuvant therapy followed by LT is still under investigation and will be completed by 2021 [218]. Regarding i-CCA, a prospective trial (NCT02878473) aiming at transplanting patients with “very early” i-CCA will confirm if these patients could benefit from transplant, which may open up a new indication for liver transplantation.

## Figures and Tables

**Table 1:** Management of PBD in perihilar cholangiocarcinoma (h-CCA).

Author, Year	PBD	N of Patients	Duration of PBD (Mean)	Overall Morbidity	Overall Mortality	Conclusions
Ferrero et al. [39], 2008	Yes No	30 30	27 days	70% 63%	3% 10%	PBD increased incidence of infectious complications
Hochwald et al. [40], 1999	Yes No	42 29	-	52% 28%	-	PBD increased incidence of infectious complications
Ramanathan et al. [41], 2018	Yes No	251 646	-	25.9% 9.3%	9.6% 3.4%	PBD is associated with increased number of bile leak and liver failure
Kennedy et al. [42], 2009	Yes No	9 12	-	0% 33%	0% 33%	PBD had a beneficial effect in patients with FLR < 30%
Farges et al. [43], 2013	Yes No	180 186	32 days	68.8% 38.8%	9.4% 11.7%	PBD does not affect overall mortality in jaundiced patients with h-CCA
Ribero et al. [44], 2016	Yes No	98 35	-	57% 49%	12% 9%	PBD is frequently complicated by cholangitis, increased risk of hepatic insufficiency, and death by liver failure

Abbreviations: FLR, functional liver remnant; N, number; PBD, preoperative biliary drain.

**Table 3:** Main studies assessing neoadjuvant chemoradiotherapy in h-CCA.

Author, Year	Study Design	N of Patients	Resectability at Presentation	Neoadjuvant Regimen	R0/Resected (%)	Outcomes
McMasters et al. [112], 1997	Retrospective	5	unresectable	5-FU + EBRT	5/5 (100)	No recurrence
Katayose et al. [113], 2015	Prospective (NACRAC study)	24	advanced, but possibly resectable	Gem + EBRT	17/21 (80.9)	mDFS and mOS yet to be determined (ongoing)
Jung et al. [114], 2017	Retrospective	12	unresectable	5-FU/Gem + EBRT	10/12 (83.3)	Downstaging: 91.7% Recurrence 83.3% mDFS 26 mo. mOS 32.9 mo.
Sumiyoshi et al. [115], 2018	Retrospective	8	unresectable	S-1 + EBRT	5/6 (83.3)	Recurrence 40% among R0

Abbreviations: EBRT, external beam radiation therapy; Gem, gemcitabine; mDFS, median disease-free survival; mOS, median overall survival; N, number; 5-FU, 5-fluouracil.

**Table 4:** Intrahepatic cholangiocarcinoma, post-operative outcomes.

Author, Year	N of Patients	Morbidity	Mortality	Median Survival (mo)	5-Year Survival
DeOlivera et al. [4], 2007	44	5%	4%	28	40%
Lang et al. [123], 2009	158	44%	7.1%	-	21%
Luo et al. [124], 2014	1333	11.5%	0.6%	30	28.7
Tabrizian et al. [125], 2014	82	-	1%	27	16%
Spolverato et al. [126], 2015	584	-	-	27	22%
Waseem et al. [127], 2017	90	-	-	15.8	11%

Abbreviations: mo, months; N, number.

**Table 6:** Results of liver transplantation for h-CCA.

Author, Year	Country	N of Patients	N of OLTs	Neoadjuvant Therapy (CT+/−RT)	Mayo Protocol (%)	1-Year OS	3-Year OS	5-Year OS
Heimbach et al. [200], 2004	United States	106	65 (61%)	Yes	0	91%	-	76%
Rea et al. [201], 2005	United States	71	38 (53.5%)	Yes	0	92%	82%	82%
Robles et al. [195], 2007	Spain	66	10 (15.1%)	No	0	80%	60%	37%
Seehofer et al. [196], 2009	Germany	16	16 (100%)	No	0	63%	-	38%
Darwish et al. [203], 2012	United States	287	216 (75%)	Yes	0	-	68% (2-year)	53%
Mantel et al. [209], 2016	Europe (ELITA database)	173	105 (60.7%)	Yes	28 (16%)	-	-	32% *
Ethun et al. [208], 2018	United States	304	70 (23%)	Yes	0	-	72%	64%
Zaborowski et al. [210], 2020	Ireland	37	26 (70.3%)	Yes	37 (100%)	81%	69%	55%
Tan et al. [198], 2020	United States	247	74 (30%)	Yes	0	84.9%	66.5%	55.6%

Abbreviations: CT, chemotherapy; N, number; OLT, orthotopic liver transplantation; OS, overall survival; RT, radiotherapy. * 59% within the «Mayo Clinic protocol».
